# Supplementary material for: Phenotypic characterization of HAM1, a novel mating regulator of the fungal pathogen Cryptococcus neoformans
Source: Microbiol Spectr. 2024 Jun 6;12(7):e03419-23. doi: 10.1128/spectrum.03419-23 (PMC11218459; doi:10.1128/spectrum.03419-23)
Supplement: Fig. S1 — Cellular fusion assays with or without exogenous pheromone addition. [file spectrum.03419-23-s0001.docx]

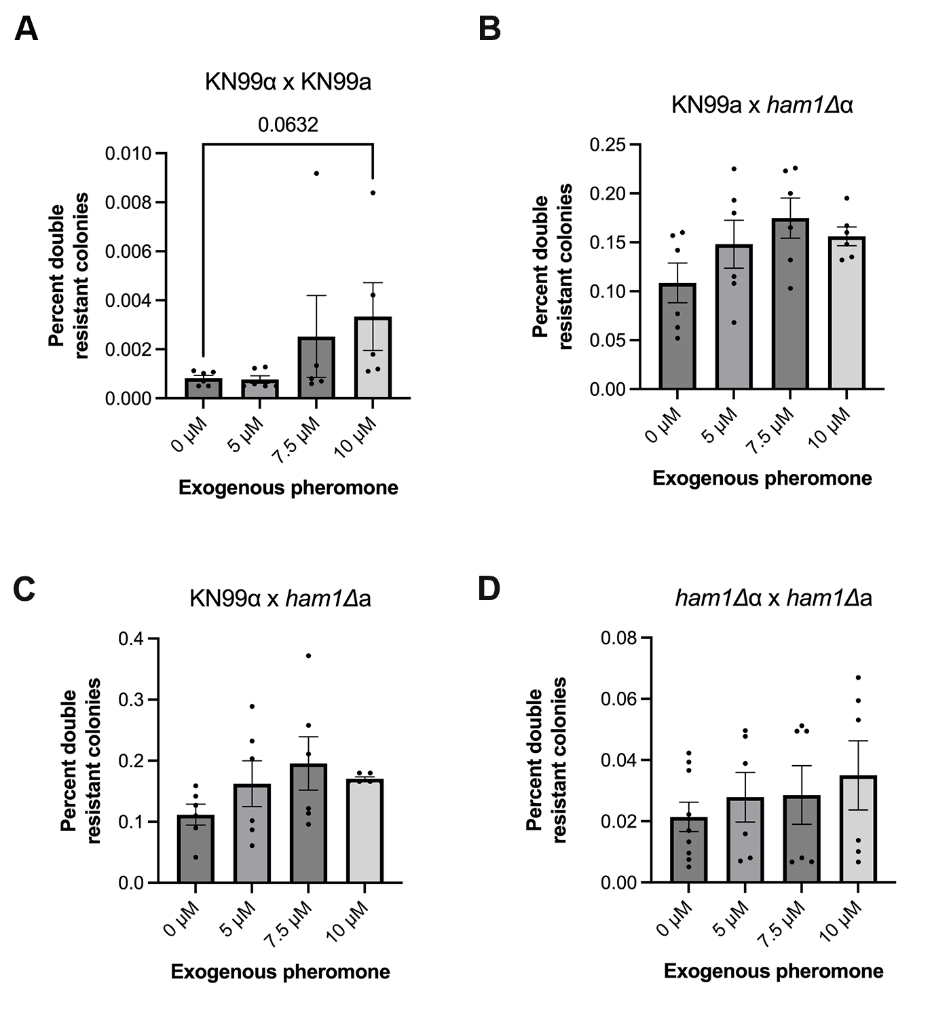


Supplemental Figure 1: **Cellular fusion assays with or without exogenous pheromone addition.** (A – D) Cell fusion results from bisexual crosses without and with 5µM, 7.5µM, and 10µM exogenous pheromone, in WT mating controls (A), *ham1*Δα unilateral cross (B), and *ham1*Δa unilateral cross (C), and *ham1*Δ mutants bilateral cross (D). Statistics are a one-way ANOVA with multiple comparisons (Kruskal-Wallis test) on all conditions, none were statistically significant. N = 5 – 9 biological replicates per condition. Values from each individual replicate are shown as black dots. The bars show the average and the standard error.
