# Supplementary material for: Phenotypic characterization of HAM1, a novel mating regulator of the fungal pathogen Cryptococcus neoformans
Source: Microbiol Spectr. 2024 Jun 6;12(7):e03419-23. doi: 10.1128/spectrum.03419-23 (PMC11218459; doi:10.1128/spectrum.03419-23)
Supplement: Fig. S2 — Commercial mutant (C07) behaves similarly to ham1Δ mutants in key mating and virulence assays. [file spectrum.03419-23-s0002.docx]

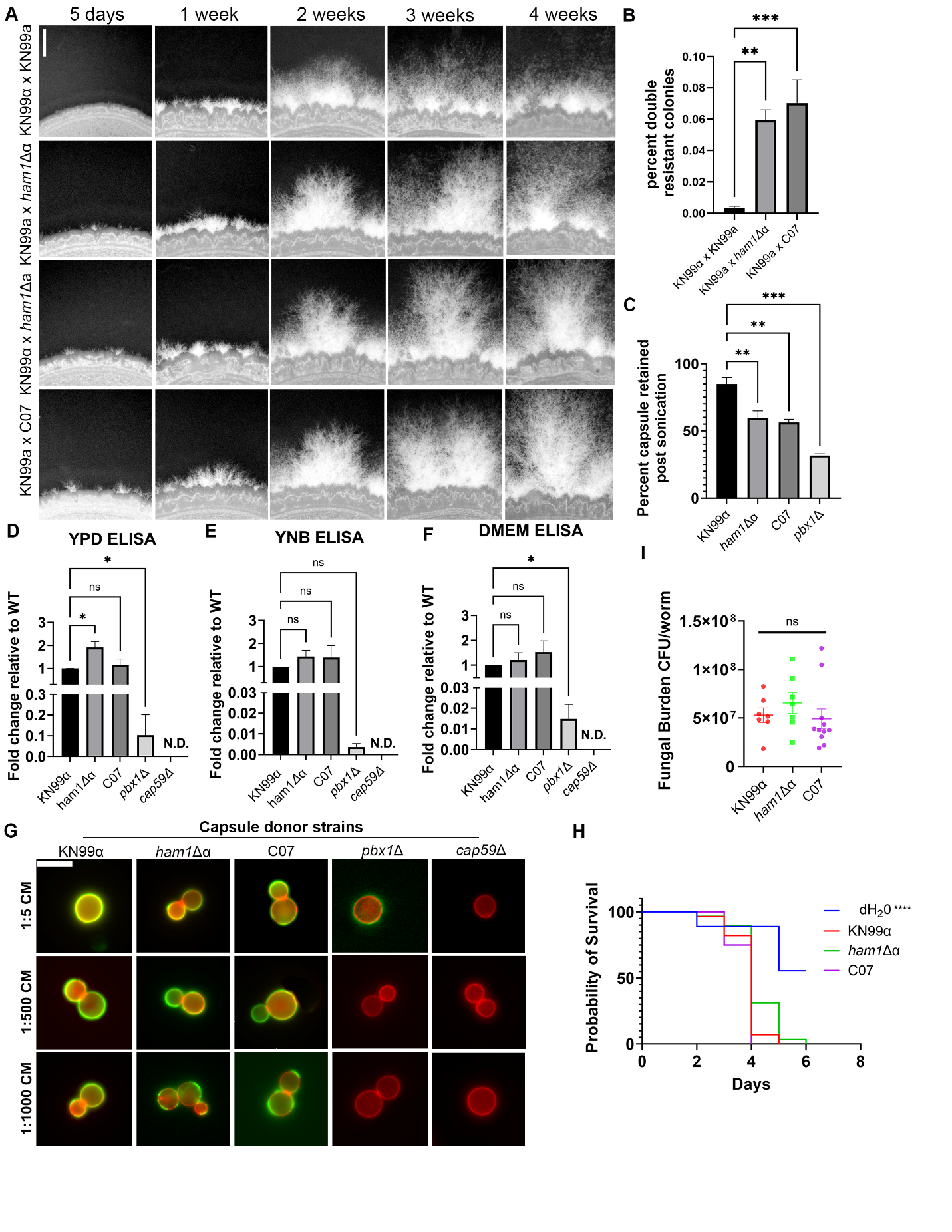


Supplemental Figure 2: **The commercial strain C07 behaves similarly to in-house *ham1*Δ mutants in key mating and virulence assays.** The most recent addition to the *C. neoformans* deletion collection contained a deletion mutant of *HAM1* which was well number C07 (https://www.fgsc.net/). We used the C07 mutant in a variety of assays together with our *ham1*Δ mutants made by biolistics, but for simplicity and clarity, we did not include C07 on the main figures. Here, we show the phenotypes present in C07 to provide external validation of the phenotypes we see in our *ham1*Δ mutants. (A) C07 produces hyphae at a similar speed and robustness to our *ham1*Δ mutants. The first 3 rows are the same images shown in Fig. 3. (B) C07 produces similar amounts of double resistant progeny to our *ham1*Δ mutants. A one-way ANOVA was run using multiple comparisons to the WT cross. ** p=0.0014, ***p=0.0003. N = 3 biological replicates for all conditions (C) C07 has similar defects in capsule attachment to *ham1*Δ mutants post sonication. A one-way ANOVA was run using a Dunnett's multiple comparison test of all mutant crosses to the WT cross; ** p=0.0062 (*ham1*Δα) **, p=0.0040 (C07), ***p=0.0004. N = 20 cells for all conditions. (D - F) Quantification of capsule shedding by C07 strain in non-capsule inducing media YPD (D) and YNB (E) and in the capsule inducing medium DMEM (F). All results were compared by one-way ANOVA with a Fisher’s LSD test, * P < 0.05. N = 3 biological replicates for all ELISAs (G) C07 fails to fully transfer capsule at high dilutions greater than 1:750. Representative images of capsule transfer capsule visualized by conjugated 3C2 antibody with Alexa488 fluorescent probe and cell wall stained with calcofluor white (CFW) scale bar is 10μm. (H) C07 shows no difference in survival compared to WT. Kaplan-Meier survival curve of dH_2_O=10 KN99α N = 30, *ham1*Δα N = 30 and C07 *N =* 30. No statistical differences were seen in the mutants but the dH_2_O survived significantly longer, **** P < 0.0001 by Mantel-Cox test. (I) C07 shows no difference in fungal burden. KN99α N = 7, *ham1*Δα *N =* 7, C07 N = 11. A one-way ANOVA with a Dunnet’s multiple comparison test of all conditions to WT was run and showed no significant difference between mutants and WT.
