# Supplementary material for: Phenotypic characterization of HAM1, a novel mating regulator of the fungal pathogen Cryptococcus neoformans
Source: Microbiol Spectr. 2024 Jun 6;12(7):e03419-23. doi: 10.1128/spectrum.03419-23 (PMC11218459; doi:10.1128/spectrum.03419-23)
Supplement: Fig. S3 — ham1Δ shows no sensitivities to various cell wall and membrane stressors as well as antifungal treatments. [file spectrum.03419-23-s0003.docx]

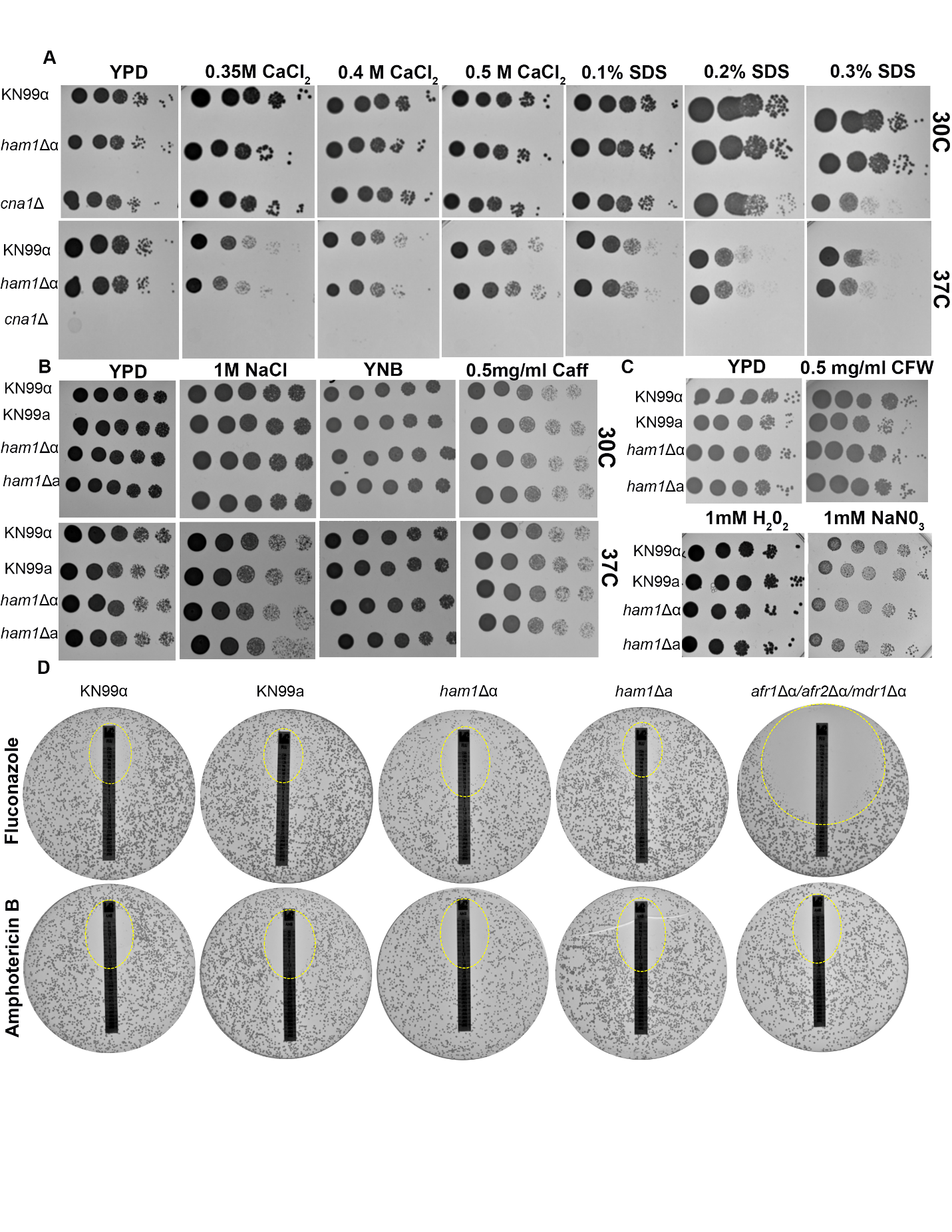


Supplemental Figure 3: ***ham1*Δ shows no sensitivities to various cell wall and membrane stressors as well as antifungal treatments.** To rule out any issues with calcineurin signaling, cell membrane, or cell wall, we tested various conditions in YPD solid agar medium. (A) Growth of indicated strains in the presence of calcium (CaCl_2_) and sodium dodecyl sulfate (SDS) at 30°C or 37°C. (B) Growth of indicated strains to test thermal stress (YPD at 30°C and 37°C), osmotic stress (NaCl), nutrient stress (YNB), and cell wall stress signaling (Caffeine). (C) Growth of indicated strains under cell wall stress (calcofluor white, CFW), oxidative stress (H_2_O_2_) and oxidative stress (NaNO_3_). (D) Growth of indicated strains in the presence of E-test strips of common antifungals (Amphotericin B and Fluconazole).
