# Supplementary material for: Phenotypic characterization of HAM1, a novel mating regulator of the fungal pathogen Cryptococcus neoformans
Source: Microbiol Spectr. 2024 Jun 6;12(7):e03419-23. doi: 10.1128/spectrum.03419-23 (PMC11218459; doi:10.1128/spectrum.03419-23)
Supplement: Fig. S4 — Ectopic complementation of ham1Δα restores the capsule shedding defect.. [file spectrum.03419-23-s0004.docx]

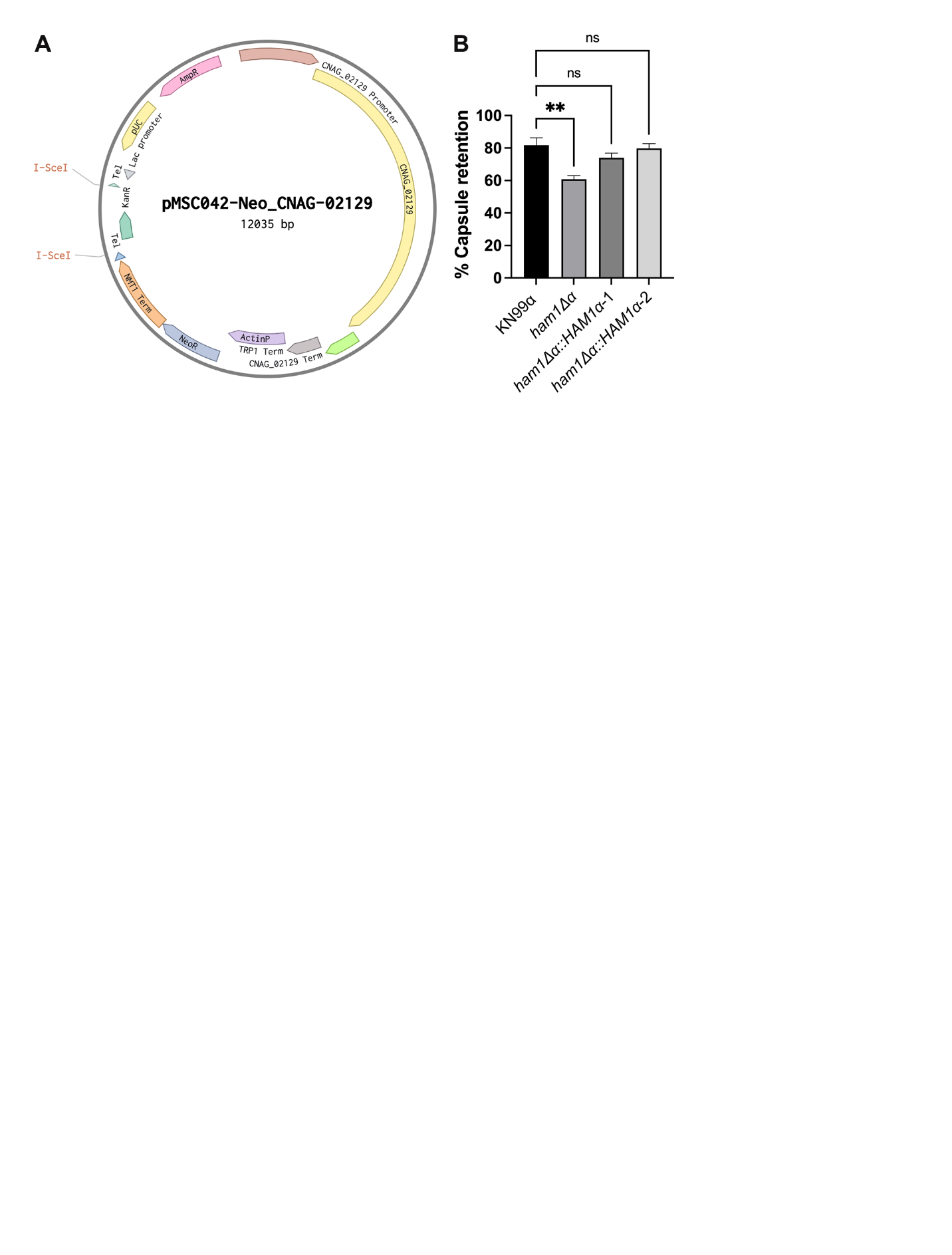


Supplemental Figure 4: **Ectopic complementation of *ham1*Δα restores the capsule shedding defect.** (A) Plasmid map used for ectopic complementation of *ham1Δ*α. This plasmid (pMSC042-Neo, a gift from the Doering lab) contains telomeric sequences flanking a small piece of the kanR gene. When linearized with I-SceI, the plasmid DNA is capped by these telomeric sequences, preventing degradation. This plasmid drives the expression of *HAM1* by its native promoter and terminator sequences. (B) Results from capsule attachment assay using sonication. Shown is 2 biological replicates combined. One-way ANOVA with multiple comparisons; ** P < 0.01.
